# Application of Quantum Dots in Chinese Herbal Medicines: Advances in Detection, Pollutant Degradation, and Pharmacological Enhancement

**DOI:** 10.3390/s25237161

**Published:** 2025-11-24

**Authors:** Delai Zhou, Fude Yang, Jian Xu, Fankui Zeng

**Affiliations:** 1 Research Center for Natural Medicine and Chemical Metrology, Lanzhou Institute of Chemical Physics, Chinese Academy of Sciences, Lanzhou 730000, China; gszyzdl2022@163.com; 2 College of Pharmacy, Gansu University of Chinese Medicine, Lanzhou 730101, China; gszyyfd@163.com; 3 Yantai Zhongke Research Institute of Advanced Materials and Green Chemical Engineering, Yantai 264006, China; 4 Qingdao Center of Resource Chemistry & New Materials, Qingdao 266100, China

**Keywords:** quantum dots, Chinese herbal medicines, active ingredients, exogenous contaminants, pharmacological effects, mechanisms

## Abstract

Quantum dots (QDs) are a class of nanomaterials with unique fluorescent properties that have gained significant attention in the research of Chinese herbal medicines (CHMs). Due to their exceptional optical characteristics, stability, biocompatibility, and other advantages, QDs are increasingly utilized in CHM studies. This review explores the diverse applications of QDs, including their use in detecting active ingredients and common exogenous pollutants in CHMs, as well as in assessing the degradation of such pollutants in both CHMs and their growing environments. Furthermore, the paper discusses the potential of QDs synthesized from CHMs as tools for analyzing other substances and modulating their pharmacological effects. The review also highlights the preparation methods, detection principles, and specific research strategies related to QDs. Integrating QDs into CHM research is poised to drive the modernization and internationalization of the CHM industry.

## 1. Introduction

In 1986, American scientist Mark Reed introduced the term “quantum dot” to describe a completely confined, zero-dimensional object. QDs are a type of quasi-zero-dimensional nanosemiconductor material, typically with dimensions less than 100 nm, most commonly ranging from 2 to 20 nm [1]. These dimensions are smaller than the exciton Bohr radius of semiconductor materials, resulting in the confinement of electrons and holes in a tiny space, forming discrete quantized energy levels [2]. When visible light is shone on QDs, photons excite electrons to higher energy levels, which then return to the stable ground state, emitting light specific to the material’s frequency. This results in narrow peaks, tunable luminescence wavelengths, broad-spectrum absorption, bright emission, and good optical stability [3]. Additionally, optimizing surface ligands and the core–shell structure enhances their chemical stability, thermal stability, and biological compatibility [4]. In recent years, QDs have gained increasing attention in biological and medical research due to their excellent optical properties and safety [5,6,7].

As an emerging nanomaterial, QDs not only serve in bioimaging and cellular labeling but can also be widely applied to detect various targets—such as tumor markers, antibiotic residues, and environmental gases—by surface modification with specific recognition elements (e.g., antibodies, molecularly imprinted polymers) [8,9,10,11]. In recent years, in particular, the performance of quantum dots has been significantly improved through multiple strategies. For instance, the design of core–shell heterostructures has greatly enhanced their fluorescence intensity and quantum yield [12]. On the other hand, advanced encapsulation and surface engineering effectively suppress surface defects and enhance stability. For instance, encapsulating CH_3_NH_3_PbBr_3_ (MAPbBr_3_) QDs within the porous zeolite imidazole framework-11 (ZIF-11) revealed that this MAPbBr_3_@ZIF-11 composite exhibits a distinctive narrow-band cyan emission at 505 nm, with a fluorescence quantum yield exceeding 85%. Notably, the composite also demonstrated outstanding solvent, thermal, ultraviolet radiation and environmental stability. This may be attributed to the framework protection and internal passivation of uniformly dispersed MAPbBr_3_ QDs, endowing them with superior optical properties and exceptional stability [13]. Moreover, the integration of quantum dots with microfluidic chips and machine vision algorithms has enabled the construction of intelligent sensing platforms, demonstrating the great potential of QDs in convenient and high-precision point-of-care testing. For instance, in a recent advancement, researchers developed a ratio-based fluorescence probe for the visual and fluorescent dual-mode detection of thiram, leveraging the partial quenching of carbon dots’ (CDs’) fluorescence by gold nanoparticles (AuNPS) and the adsorption of thiram onto the nanoparticles. Utilizing 3D printing technology and smartphone sensing devices, they subsequently engineered a corresponding portable detection apparatus [14].

As a treasure of Chinese civilization, CHMs have become the focus of efforts aimed at modernization and internationalization [15]. Among the key challenges are effective quality control and the high-quality utilization of resources [16]. The integration of active ingredients analysis with strategies for controlling exogenous contaminants has become a common tool in CHM quality control [17,18]. Additionally, the concept of quality markers has been developed and is gaining scientific support for numerous Chinese medicinal products [19]. The continuous development of exogenous contaminant detection technologies plays a crucial role in the quality control of CHMs [20]. Conventional methods such as chromatography, mass spectrometry, and spectral analysis are widely used in pharmacopoeias worldwide to quantify active ingredients and exogenous pollutants in CHMs. However, these methods have limitations, such as high testing costs, long analysis times, and lack of portability [21].

**Figure 1:**
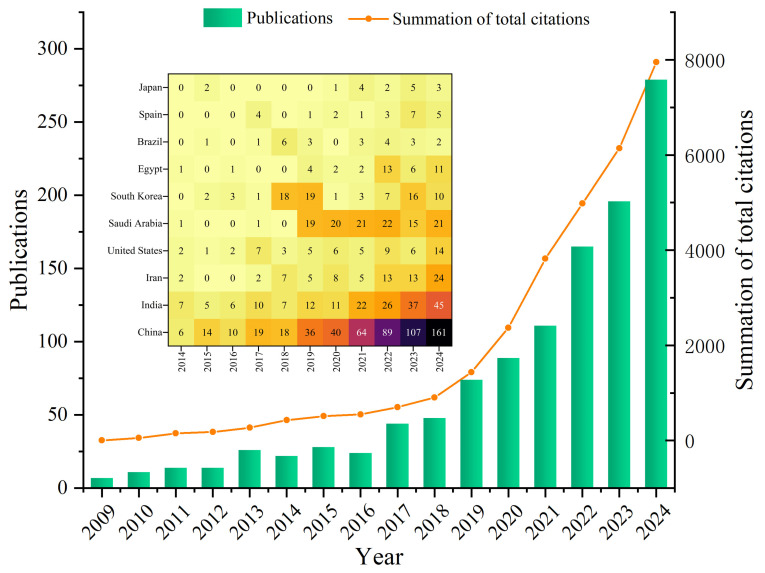
Articles on the application of QDs in CHMs from “web of science” statistical data.

Recently, with the rapid advancement of nanomedicine, the use of natural substances as raw materials for nanomaterials has gained popularity. After considering requirements for low toxicity and clinical safety, CHMs and their extracts have become important precursors for carbon quantum dots (CQDs) [22]. CHM-derived CQDs not only exhibit diverse biological activities but also possess excellent optical properties that can be used to construct fluorescent sensors [23]. As a result, QDs have become increasingly integrated into CHM research. In this study, searching QD-related CHM studies in the “Web of Science” reveals that from 2009 to 2024, the interest in QDs within CHM research has been steadily increasing, with the number of publications also rising, as shown in Figure 1. This paper focuses on reviewing research reports concerning CHM-QDs published over the past decade.

In this paper, we review recent reports on the application of QDs in CHM research. We focus on diverse QD applications in the detection of active ingredients and exogenous pollutants, the degradation of environmental pollutants in CHMs, and the use of CHM-derived QDs as materials for further research. We also discuss material preparation methods and research design strategies in these specific applications, providing a reference for future explorations of QDs in CHM research.

## 2. Application of QDs in the Detection of Active Ingredients in CHMs

The active ingredients of herbal medicines are the chemicals that are responsible for their therapeutic or prophylactic effects. These constituents, through their pharmacological activities, produce defined physiological responses in the human body [24]. The active ingredients in CHMs are the primary contributors to their pharmacological effects. Currently, the majority of international quality standards for CHMs rely on quantitative analysis of these active ingredients to ensure product quality. For example, the Chinese Pharmacopoeia sets specific limits for the active ingredients found in herbs [25]. CDs have become a popular tool for analyzing the active ingredients in CHMs [26]. Guo et al. [27] synthesized nitrogen-doped carbon quantum dots (N-CQDs) using a mixed solvent system of tetraethylene glycol and water. These N-CQDs were found to be quenched by phenolics, such as chlorogenic acid, salvianolic acid B, and rutin. The researchers further developed an N-CQD-based paper testing device, which could collect color data using a smartphone. This method has been demonstrated to be effective for determining the total phenolic content in honeysuckle extracts.

A common approach to improving the optical properties of QDs is modification. Xu et al. [28] prepared histidine and pentaethylenehexylamine-functionalized boron-doped graphene quantum dots (HPB-GQDs) via the pyrolysis of a mixture containing citric acid, histidine, pentamethylhexamine, and boric acid. These HPB-GQDs exhibited a fluorescence quantum yield of up to 87.4%, which is superior to that of both graphene quantum dots (GQDs) and GQDs functionalized with single components like histidine, pentaethylenehexamine, or boric acid. Using the fluorescence quenching effect of curcumin on HPB-GQDs, a fluorescence-based method was established to determine the curcumin levels in CHMs.

One significant challenge in fluorescence sensing systems is the identification of substances. To address this, researchers have developed a dual-template molecularly imprinted, dual-emission ratio fluorescent sensor designed for the detection of methyl eugenol (ME) and aristolochic acid (AA). The sensor utilizes ME and AA as template molecules to prepare perovskite QD molecularly imprinted polymers (MIPs). These compounds exhibit different fluorescence burst effects at 515 nm and 650 nm, allowing for qualitative identification based on the color emitted. Moreover, the concentration-dependent color changes enable the semi-quantitative detection of ME and AA [29].

Fluorescence detection strategies using QDs have also proven effective in distinguishing between different quality grades of CHMs. It is widely recognized that the active ingredients in CHMs can vary significantly in quality, particularly in the context of “Daodi” CHMs [30]. Long et al. [31] developed a fluorescent array sensor based on gold nanoclusters (AuNCs) and N-acetyl-L-cysteine (NAC)-modified cadmium telluride quantum dots (CdTe QDs). The sensor utilizes hydrogen bonds between amino acids in lilies and polyvinylpyrrolidone on the surface of AuNCs, causing a blue shift in the fluorescence spectrum. Additionally, the protons released from phenolic acids and amino acids cause NAC to transition from an ionic to a molecular state, weakening the charge repulsion between QDs and triggering an aggregation-induced fluorescence enhancement effect. Due to the varying contents of amino acids and phenolic acids in lily bulbs from different sources, the sensor array produces distinct fluorescence patterns. Combined with pattern recognition through a random model, the sensor achieved a prediction accuracy of 94.4% for identifying the source of the lily bulbs.

Table 1 provides a concise summary of the major types of QDs employed for detecting active ingredients in CHMs, illustrating the versatility and innovation of QD-based sensing platforms. CDs and their doped derivatives are the most widely applied, primarily due to their low toxicity, straightforward synthesis, and biocompatibility. Their fluorescence responses, typically mediated by PET or the IFE, have been extensively exploited for detecting flavonoids such as hyperin, baicalein, morin, and myricetin, with detection limits reaching nanomolar levels. These findings highlight the sensitivity of QD-based sensors in discriminating structurally related flavonoids, which are key pharmacologically active constituents of CHMs. Moreover, heteroatom doping (e.g., nitrogen, sulfur) has proven effective in enhancing quantum yield and fine-tuning selectivity, thereby broadening the applicability of CDs in CHM quality assessment.

Importantly, a range of engineered QD systems further expands analytical capabilities. The integration of MIPs with QDs provides a dual advantage of molecular recognition and fluorescence signaling. For example, MIP-modified Mn-doped ZnS QDs and FeS_2_ QDs enabled the specific recognition of complex molecules such as celastrol and aconitine, achieving detection limits as low as 24 nM. This coupling strategy demonstrates a promising avenue for targeting pharmacologically potent but structurally challenging compounds in CHMs. Aptamer-modified QDs, such as those designed for beclomethasone detection, illustrate the utility of biomolecular recognition to enhance specificity and reduce interference from complex herbal matrices. Hybrid sensing platforms, including polyaniline-functionalized GQD electrodes, integrate optical and electrochemical modalities to improve electron transfer efficiency, underscoring the multifunctional potential of QDs. Ratiometric fluorescent probes also represent an emerging strategy, minimizing environmental interference and improving accuracy, as demonstrated in the detection of aristolochic acid I. Collectively, these advances indicate that QDs probably accelerate the development of next-generation sensors, thereby contributing to more reliable quality control into CHMs.

## 3. Application of QDs in the Detection of Common Exogenous Pollutants in CHMs and Related Environments

### 3.1. Application of QDs in Pesticide Residue Detection

The extensive use of pesticides in various fields such as agricultural production, environmental protection, and household hygiene, as well as in preventing mold and pests in industrial products, has brought about widespread potential threats to agricultural products, water sources, and soil. Traditional Chinese medicine is no exception. Multiple studies have shown that the health risks posed by pesticide residues in traditional Chinese medicine cannot be ignored. Therefore, developing convenient and rapid detection methods is of great significance for the flexible and comprehensive prevention and control of pesticide residue pollution [43,44].

**Figure 2:**
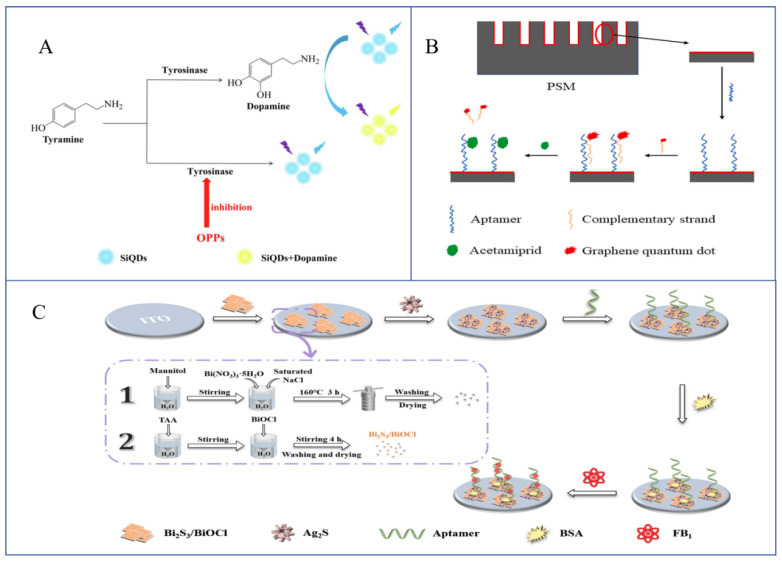
(**A**) The detection mechanism of SiQDs for methyl parathion [46]. (**B**) A method for detecting acetylaminopyridine based on an aptamer recognition strategy [54]. (**C**) Construction strategy and detection mechanism of FB1 photoelectrochemical (PEC) aptamer sensors based on Bi_2_S_3_/BiOCl composite materials [61].

Researchers have exploited the unique optical properties of QDs to develop an array of strategies for the sensitive detection of pesticide residues in CHMs and their environments. Several studies have explored sensing mechanisms that utilize enzyme inhibition by pesticide residues. Mu et al. [45] modified GQDs with glutathione and, based on fluorescence resonance energy transfer (FRET) between the QDs and Fe^3+^, successfully detected flytoxin in *Angelica sinensis*. The method involved the inhibition of acetylcholinesterase (AChE) by flytoxin phosphorus and the oxidation of Fe^2+^ by H_2_O_2_, generated from acetylcholine decomposition. A study prepared silicon quantum dots (SiQDs) that emit blue fluorescence at 440 nm. In their study, dopamine, catalyzed by tyrosinase in the presence of tyrosine, interacted with SiQDs, altering their fluorescence emission. However, organophosphorus pesticides inhibited tyrosinase activity, preventing dopamine formation. Using this strategy, they developed a method for determining the methyl parathion levels in potato and water samples, as shown in Figure 2A [46].

QDs often possess a variety of chemical groups on their surface, which interact with pesticide residues, forming a sensing mechanism [47]. For example, the ether group in cypermethrin forms an ester adduct with the carboxylate terminus of mercaptoacetic acid. This reaction rearranges to form an ester [48]. Researchers used mercaptoacetic acid-modified zinc sulfide-doped Mn quantum dots (TGA@Mn-ZnS-QDs) to detect cypermethrin. The fluorescence of TGA@Mn-ZnS QDs is quenched in the presence of cypermethrin, allowing for its detection in various environmental and agricultural samples [49]. Lin et al. [50] incorporated dithizone (DZ) into the construction of CdTe@ZnS QDs. In this system, the excess Cd^2+^ on the surface of the QDs formed coordination compounds with DZ under alkaline conditions, leading to FRET and fluorescence quenching. However, when chlorpyrifos was added, it decomposed rapidly into diethyl thiophosphate and 3,5,6-trichloro-2-pyridinol, which replaced the DZ on the surface of CdTe@ZnS QDs, disrupting the FRET mechanism and restoring fluorescence. This strategy enabled the detection of chlorpyrifos in agricultural products, as well as other pesticides such as malathion, butocarbazone, and nitenpyram [51,52,53].

To enhance detection specificity, some studies have modified QDs with aptamers for pesticide residue detection. For instance, researchers immobilized the acetamiprid aptamer in a functionalized porous silicon microcavity, then hybridized it with a complementary chain of the aptamer using GQD modification. The interaction between acetamiprid and the aptamer caused the separation of the aptamer from the complementary chain, which led to a decrease in the effective refractive index and a blue shift in the center wavelength of the reflectance spectra. This principle was used to develop a detection method for acetamiprid. The detailed detection principle is shown in Figure 2B [54]. Another study established a fluorescent aptasensor for carbendazim detection, based on the aptamer-mediated quenching of carbon dots (CDs) by AuNPs [55].

The combination of multiple QDs has been widely utilized as a signal amplification strategy in the construction of ratio-fluorescence probes. Liu et al. [56] synthesized a dual-signal ratiometric fluorescent probe consisting of mesoporous silica-encapsulated CQDs and L-cysteine-modified manganese-doped ZnS-QDs via Steglich esterification. This probe was used for detecting nicosulfuron in environmental samples. Similarly, Xiang et al. [57] developed a ratiometric fluorescent probe based on blue carbon dots (bCDs) and red quantum dots (rQDs) to quantitatively detect glyphosate in agricultural products such as potatoes, ginger, and apples. In this probe, rQDs (640 nm) served as the response signal, while bCDs (440 nm) acted as the reference signal.

The fluorescence changes in some QDs are visible to the naked eye, enabling their further development into simple and practical visual detection methods. For example, researchers constructed a fluorescent paper sensor based on N-CQDs to detect the pesticide methyl thiophanate in vegetables. In this system, fluorescence changes in N-CQDs after exposure to methyl thiophanate were recorded and analyzed using a smartphone in combination with ImageJ software, demonstrating a rapid and low-cost strategy for on-site monitoring [58]. Beyond visual detection, QDs have also been employed to construct electrochemiluminescent (ECL) sensors for pesticide residue analysis in CHMs and related environments, owing to their efficient ECL properties. Gu et al. [59] synthesized a QD-functionalized metal–organic framework composite and developed a highly sensitive ECL enzyme biosensor for detecting profenofos. The detection mechanism was based on the excellent ECL performance of the composite material, coupled with the inhibitory effect of organophosphorus pesticides on AChE activity.

In addition to enzyme-dependent strategies, efforts have been made to construct enzyme-free electrochemical sensors to improve robustness and stability. Shan et al. [60] developed a solid-state ECL sensor by immobilizing CQDs on the electrode surface, using electrospun carbon nanofibers as carriers and a simple drop-coating method for surface modification. The prepared sensor exhibited high sensitivity and accuracy in detecting malathion residues in fruits and vegetables, demonstrating the feasibility of enzyme-free electrochemical sensing systems. Collectively, these advances highlight the versatility of QDs in enabling diverse detection platforms, ranging from visual and smartphone-assisted assays to ECL biosensors, which not only expand the scope of pesticide residue monitoring in CHMs but also pave the way for portable, cost-effective, and highly sensitive analytical technologies.

### 3.2. Application of QDs in Heavy Metal Detection

Heavy metals are common environmental pollutants that, when ingested in excess, can cause severe health risks. Traditional detection techniques are often constrained by their reliance on bulky instrumentation and high operational costs, which limit their practicality in routine CHM analysis [62]. In this context, QD-based sensors offer a more convenient and versatile alternative. Zhao et al. [63] prepared N-doped carbon dots (NCDs) via hydrothermal synthesis and combined them with a nucleic acid aptamer (CD-4). The aptamer could selectively bind Cd^2+^ through electrostatic interactions, leading to fluorescence quenching, which was successfully applied to the detection of Cd^2+^ in *Panax notoginseng*. Similarly, He et al. [64] synthesized core–shell structured CDs, in which the peripheral carboxyl groups facilitated aggregation with Hg^2+^, promoting electron transfer from the CDs to Hg^2+^ and effectively quenching fluorescence. This strategy was applied to detect Hg^2+^ in proprietary Chinese medicine samples.

Beyond CDs, other types of QDs have also been explored. For example, Li et al. [65] synthesized sulfur quantum dots (SQDs) with excellent stability and water solubility using a one-pot hydrothermal method, exploiting the IFE between SQDs and Co^2+^ to detect cobalt ions in *Salvia miltiorrhizae* Radix et Rhizoma. Similar approaches have been applied to copper detection: glutathione-modified CdTe QDs were used as fluorescent probes, where Cu^2+^ induced aggregation-caused quenching via interactions with surface carboxyl groups. To reduce reliance on sophisticated instruments, a partial least squares regression (PLSR) model was incorporated into the detection strategy, enabling Cu^2+^ quantification through colorimetric principles [66]. Given the complexity of heavy metal contamination in CHMs and related environments, multi-QD strategies have also been developed. Wang et al. [67] designed a three-channel fluorescence array sensor integrating AuNCs, NCDs, and AuNCs@NCDs to simultaneously detect Cd^2+^, Pb^2+^, and Hg^2+^ in water, soil, and CHMs. By combining colorimetric responses with PLSDA discrimination and PLSR recognition, the system allowed for accurate visualization and quantification in complex samples, and further proposed a tunable fluorescence spectral logic device adaptable to different regulatory standards.

### 3.3. Application of QDs in Mycotoxin Detection

Mycotoxins are another major class of exogenous contaminants in CHMs, posing significant safety concerns. A study conducted a large-scale analysis of 35 fungal toxins in 60 commonly used CHMs in the Chinese market. The results showed that among the 60 CHMs, 50 were contaminated by mycotoxins (accounting for 83.3% of the incidence rate), with the contamination range of the strains being 1.7–48.0 μg/kg. Mycotoxins such as aflatoxin, chaetoglobosin A, gliotoxin, etc., were widely detected [68].

Nucleic acid aptamers are the most common recognition elements used in QD-based mycotoxin detection. For instance, fungal toxin aptamers can form FRET systems with graphene oxide (GO), resulting in fluorescence quenching. When the aptamer specifically binds to mycotoxins, the probe is released from the GO surface, partially restoring fluorescence. Based on this “on–off–on” mechanism, a fluorescent aptasensor was developed for the detection of aflatoxin A and zearalenone (ZEN) in Coicis Semen [69]. Similarly, a CdTe QD-based fluorescent sensor was constructed for ochratoxin detection in Astragalus [70]. Beyond fluorescence, QDs have also been integrated into electrochemical platforms. A PEC aptasensor was designed using a Bi2S3/BiOCl composite material sensitized with in situ grown Ag_2_S QDs, enabling the highly sensitive detection of FB1 (Figure 2C) [61]. Comparable strategies have been extended to ZEN detection [71].

**Figure 3:**
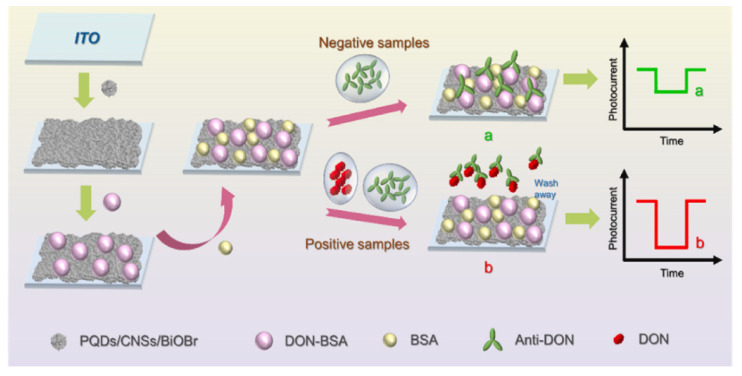
Mechanism of DON detection by PQDs@CNSs@BiOBr [73].

In addition to aptamers, antibodies can also serve as recognition elements in QD-based sensors. Kong et al. [72] developed a label-free ECL immunosensor for aflatoxin B1 (AFB1) detection in lotus seeds. The system employed ZnCdS@ZnS QDs as ECL probes immobilized on Au electrodes and anti-AFB1 antibodies as capture agents. Chen et al. [73] further enhanced QD-based PEC immunosensing by decorating carbon nanospheres with methylamine-brominated chalcogenide QDs (PQDs@CNSs), which improved the photoelectric activity of BiOBr via heterojunction formation. The ternary PQDs@CNSs@BiOBr composites were coated on indium tin oxide (ITO) electrodes and functionalized with deoxynivalenol–bovine serum albumin conjugates, enabling the sensitive detection of deoxynivalenol (DON) (Figure 3). Collectively, these studies highlight the potential of QDs in developing both aptamer- and antibody-based platforms for mycotoxin monitoring, providing versatile and highly sensitive strategies for ensuring the safety of CHMs.

### 3.4. QD Applications in the Detection of Other Exogenous Pollutants

In addition to the commonly studied contaminants such as heavy metals, pesticide residues, and mycotoxins, QDs have also been applied to the detection of other exogenous pollutants that, although less frequently reported, are of growing concern in CHMs. For instance, chronic exposure to plasticizers has been linked to reproductive and immune dysfunction, making them an emerging risk factor in CHM safety [74]. Researchers dispersed boron-doped GQDs in solvents of differing polarity (N,N-dimethylformamide and cyclohexane) to construct a 3 × 2 fluorescence array. By selecting three characteristic fluorescence peaks of boron-doped GQDs in each solvent, the array exploited plasticizer-induced dispersion effects and corresponding fluorescence changes, enabling the detection of six different real-world samples [75]. Similarly, sulfur fumigation—a traditional processing technique for CHMs that prolongs shelf life—has raised concerns due to potential sulfite overexposure. To address this, dual-emission N-doped carbon dots (CECDs) were synthesized, and a ratiometric fluorescence sensor was developed based on the quenching of CECDs by Cr(VI) at 440 nm and the synergistic interaction between Cr(VI)/Cr(III) and HSO_3_^−^. The introduction of HSO_3_^−^ significantly enhanced the fluorescence intensity ratio (I_440_/I_500_), providing a reliable method for sulfite detection [76]. Additional QD-based fluorescent sensors have also been reported for detecting hypochlorite, bisulfite, and sulfur-containing gases in vivo, broadening the scope of environmental and CHM safety applications [77,78].

Antibiotics represent another class of exogenous pollutants of concern, particularly in animal-derived herbal medicines where their misuse during breeding poses risks to consumer health [79]. To monitor antibiotic residues, researchers developed a magnetic quantum dot material combining CdTe QDs with magnetic nanoparticles (MNPs). The resulting composite (MNP-SiO_2_-QDs) maintained high fluorescence retention and reusability, while its fluorescence could be quenched by antibiotics such as enrofloxacin, ceftiofur, doxycycline, and chloramphenicol. This platform enabled the simultaneous quantitative detection of the four antibiotics within 5 min, independent of the sample substrate [80]. Furthermore, microbial contamination remains a critical challenge for CHM quality and safety. QDs have been functionalized to construct biosensors capable of rapid microorganism detection. For example, Santos et al. [81] electropolymerized polypyrrole (PPY) on a flexible ITO electrode, introduced GQDs into the PPY layer, and further modified the surface with concanavalin A (Con A), a glucose/mannose-specific lectin with high binding affinity for fungal and bacterial cell walls. This biosensor successfully detected common pathogens including *Candida tropicalis*, *Staphylococcus aureus*, and *Escherichia coli*, based on electrochemical changes induced by Con A–microbe recognition complexes. Collectively, these studies highlight the expanding applications of QDs beyond traditional pollutants, underscoring their potential in safeguarding CHMs from diverse and emerging safety risks.

### 3.5. Analysis of QD Detection Strategies for Exogenous Contaminants in CHMs

In the preceding discussion, researchers constructed recognition strategies through diverse modifications of quantum dots, based on enzyme inhibition, direct interaction, or indirect interaction. We summarize the primary recognition mechanisms discussed above, as shown in Table 2.

As shown in Table 2, QD-sensing technology demonstrates considerable potential in contaminant detection owing to its high sensitivity and design flexibility. Systematic analysis of various sensing mechanisms provides valuable insights into their design principles and development trends. The diversity of sensing strategies represents a fundamental characteristic of this field. As summarized in Table 2, detection mechanisms span from highly specific biological recognition (e.g., aptamers, antibodies) to universal physicochemical interactions (e.g., direct coordination, dispersion regulation). This methodological diversity enables researchers to select optimal pathways according to target characteristics, achieving customized detection schemes. In the application of biological recognition elements, the coexistence of high specificity and stability challenges remains noteworthy. Despite the exceptional recognition capabilities demonstrated by biosensors, the immobilization difficulties and activity preservation of biological molecules continue to pose practical limitations. In contrast, direct interaction-based sensors (e.g., CDs for Hg^2+^ detection), while featuring simple preparation and rapid response, often face selectivity limitations that restrict their application in complex sample matrices.

Innovations in signal transduction mechanisms have substantially enhanced detection performance. The competitive binding/FRET strategy for chlorpyrifos detection, for instance, achieves signal amplification through FRET disruption and fluorescence recovery. Furthermore, strategies incorporating multiple fluorescence quenching mechanisms (such as the synergistic effect of FRET and electron transfer) have demonstrated broader linear ranges and enhanced sensitivity in multiplex detection applications. The synergistic design of materials and mechanisms proves crucial for advanced sensor development. Representative examples include N-CDs for enhanced Cd^2+^ recognition and B-GQDs utilizing dispersion changes for phthalate detection, collectively demonstrating the sophisticated integration between quantum dot surface engineering and sensing mechanisms.

Future research should prioritize the development of novel stable recognition elements, intelligent response interfaces, and portable devices to enable the on-site rapid detection of environmental and herbal medicine contaminants—a research direction requiring urgent exploration.

## 4. Application of QDs in the Photodegradation of Exogenous Pollutants and Their Prospects in CHMs

**Figure 4:**
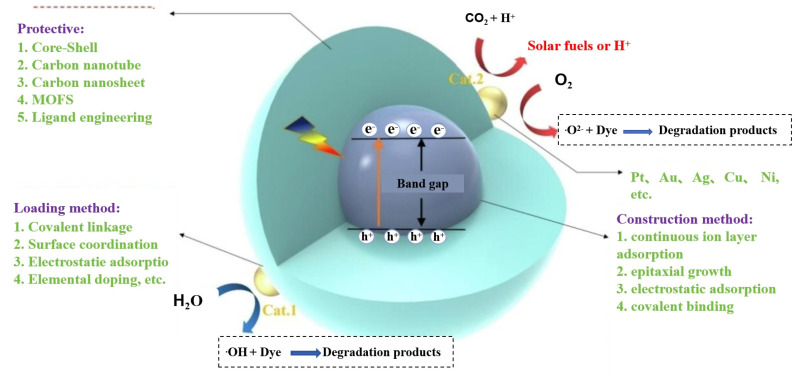
Reaction mechanism of QD photocatalytic system [82].

Photocatalysis is an emerging technology with great potential to address both environmental pollution and the energy crisis. In recent years, semiconductor quantum dots have attracted significant attention as photocatalysts owing to their exceptional visible-light absorption, multi-exciton effect, pronounced surface reactivity, and tunable bandgap structures [82,83]. As illustrated in Figure 4 [82], the catalytic efficiency of QDs can be further enhanced by loading them with suitable co-catalysts through different modification strategies. Such QD-based photocatalytic systems have demonstrated promising applications in the degradation and control of pollutants in CHMs and their cultivation environments.

Current studies have reported the photocatalytic removal of a wide range of pollutants—including pesticide residues, antibiotics, organic dyes, and other hazardous compounds—indicating the potential of QDs in improving both CHM quality and the ecological sustainability of its production processes. Importantly, “green” QDs derived from natural sources also display strong photocatalytic performance. For example, GQDs synthesized from rice husks markedly enhanced the visible-light-driven degradation of dichlorodiphenyltrichloroethane and cypermethrin, achieving removal rates of 60–70% [84]. Similarly, the catalytic activity of traditional photocatalytic materials can be significantly improved when modified with QDs. Pure g-C_3_N_4_ nanosheets degraded only 24% of sulfamonomethylpyrimidine (SMZ) under identical light conditions, whereas bisphenol S doping increased the degradation rate to 40%. Remarkably, further modification with boron nitride quantum dots (BNQDs) achieved over 97% removal of SMZ, underscoring the synergistic enhancement achievable through QD modification [85].

Despite these advances, QD-based pollutant degradation technologies remain underutilized in CHM-related applications. Developing environmentally friendly and efficient QD-based methods could significantly improve the safety and quality of CHM, while simultaneously benefiting its cultivation and processing environments. Interestingly, CHM itself can also serve as a precursor material for synthesizing QDs. For instance, Zhang et al. [86] prepared cost-effective and low-toxicity CDs from CHM residues, which not only enabled Fe^3+^ detection but also demonstrated the efficient degradation of indigo carmine dye. Likewise, Alisma was employed as a raw material to synthesize CQDs capable of completely degrading malachite green within 4.5 h under dark conditions, while also being applicable for HeLa cell imaging [87]. These examples demonstrate a dual benefit: the valorization of CHM by-products into functional nanomaterials and the development of novel QDs with pollutant remediation capability. With the continuous expansion of QD research in CHMs, it is reasonable to anticipate that QD-based photocatalytic systems will play an increasingly important role in pollutant treatment, thereby promoting the modernization, sustainability, and safety of the CHM industry.

This paper summarizes some applications of QDs in pollutant degradation, as shown in Table 3. A prominent theme from Table 3 is the growing emphasis on carbon-based “green” QDs for the photocatalytic degradation of pollutants associated with CHMs. CDs, GQDs, and their doped derivatives are increasingly synthesized from low-cost, renewable biomass such as citrus peels or CHM residues, reflecting a convergence of waste valorization and environmental remediation. These materials exhibit excellent visible-light responsiveness and biocompatibility, enabling the efficient degradation of diverse pollutants, including pesticides and dyes. For example, N-CDs derived from citrus peels achieved 91% degradation of chlorpyrifos under natural daylight conditions, demonstrating their potential for solar-driven applications. Similarly, CDs synthesized from herbal residues were used for the degradation of synthetic dyes such as indigo carmine, showing that CHM by-products themselves can be transformed into functional nanomaterials. Beyond pesticides and dyes, green QDs have also been integrated into advanced systems, such as photo-Fenton catalysts, enabling antibiotic degradation (e.g., ciprofloxacin removal of 76%) under visible light. Collectively, these findings highlight that sustainable synthesis routes not only address the safety challenges of CHMs but also provide scalable, eco-friendly photocatalysts, aligning with the broader goals of a circular economy and green nanotechnology.

In parallel, hybrid and doped QDs offer superior catalytic efficiency by overcoming the intrinsic limitations of traditional photocatalysts. Non-metal and metal doping strategies, as illustrated by BNQDs combined with bisphenol S-doped g-C_3_N_4_ nanosheets, achieved 100% removal of sulfadimethoxine antibiotics within 60 min, far surpassing unmodified g-C_3_N_4_. Similarly, Cu-doped CQDs loaded onto Ni-MOF platforms enhanced charge separation and visible-light absorption, reaching 93.5% degradation of tetracycline. Metal chalcogenide QDs such as CdS, CdTe, and ZnSe have also demonstrated high degradation efficiencies (70–99%) for pesticides and dyes, though their potential toxicity and long-term environmental risks remain concerns. Notably, QD composites like ZnS-SGQDs or CDBHCF nanocomposites have achieved the near-complete degradation of complex pollutants, indicating that synergistic coupling with semiconductors or metal–organic frameworks is an effective strategy for expanding pollutant selectivity and robustness. At the same time, applications have extended beyond conventional CHM contaminants to broader organic pollutants, such as octane, underscoring the versatility of QD-based photocatalysis. Taken together, Table 3 demonstrates that while carbon-based QDs represent the most sustainable pathway for CHM pollutant remediation, hybrid and doped QD systems currently deliver the highest efficiencies. Future research should focus on integrating the sustainability of green QDs with the performance advantages of hybrid systems, while addressing challenges related to scalability, long-term stability, and biosafety, to fully harness QDs as next-generation tools for CHM quality assurance and environmental management.

## 5. Preparation and Application of QDs Made from CHMs

### 5.1. Applications in Detection and Analysis

CHMs are rich in cellulose, lignin, hemicellulose, and diverse chemical constituents. These features—simple extractability, high yield, abundant functional groups, and the presence of natural impurities—make CHM and its by-products ideal precursors for synthesizing CDs. Leveraging these advantages, Zhang et al. [98] synthesized N,Cl-doped CDs through a one-step solvothermal approach using waste CHMs as the carbon precursor. The resultant CQDs demonstrated unique triple-emission fluorescence characteristics and enabled the sensitive detection of Cr(VI) via dynamic fluorescence quenching across three emission channels. In a similar manner, N,S,B co-doped carbon nanodots (CNDs) were fabricated via a hydrothermal method using waste Clematis chinensis as the carbon source, supplemented with urea, thiourea, and boric acid as nitrogen, sulfur, and boron precursors, respectively. These doped CNDs were successfully employed for the detection of furazolidone in food samples [99].

**Figure 5:**
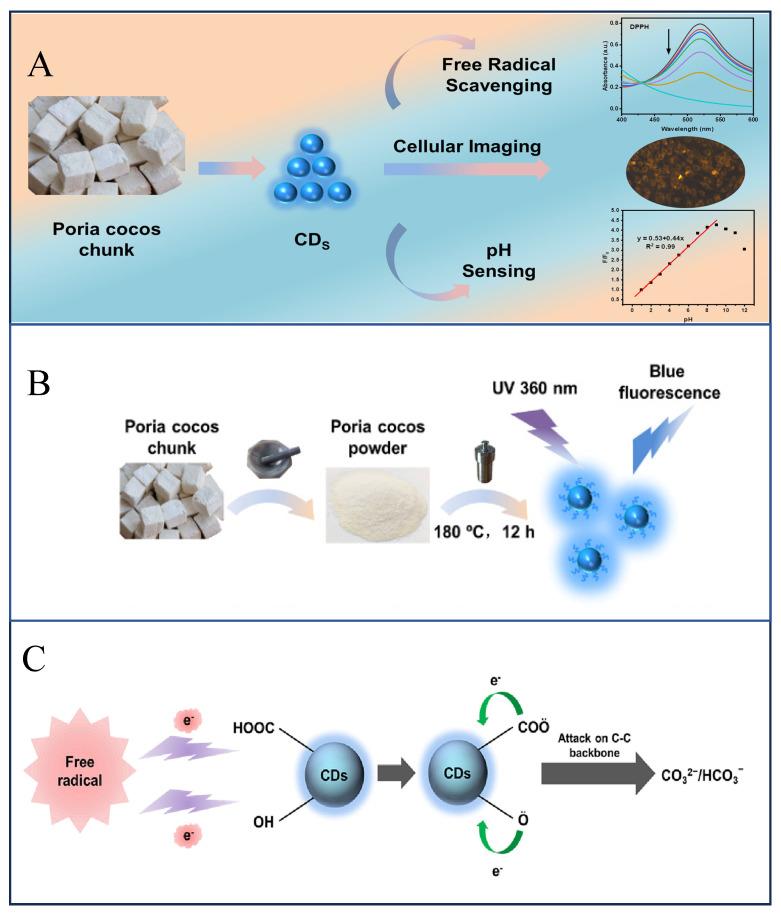
(**A**) Strategies for the application of CDs prepared from Poria cocos in cellular imaging, free radical scavenging, and pH determination. (**B**) Preparation strategies for CDs. (**C**) Free radical scavenging mechanism [104].

Other CHM-derived CDs have been exploited for “off–on” fluorescence strategies. Li et al. [100] synthesized CDs from the residues of Gardenia jasminoides after medicinal use, demonstrating that their fluorescence could be quenched by Ag^+^ and subsequently restored by S^2–^, enabling a selective and sensitive method for sulfide detection in water. Several studies have further confirmed that CHM wastes can be used to produce functional CDs capable of detecting both metal and non-metal ions in complex environmental matrices [101,102,103]. Beyond pollutant sensing, CHM-derived CDs also exhibit excellent biocompatibility. For example, CDs prepared from Poria cocos displayed high water dispersibility, strong photoluminescence, and low cytotoxicity. These properties enabled the multicolor bioimaging of MDA-MB-435S cells. Moreover, these CDs showed notable free radical scavenging activity and reversible luminescence responses to pH changes, which were further exploited to construct a highly sensitive pH sensor (Figure 5) [104]. Collectively, these studies demonstrate that CHM not only serves as a renewable resource for producing eco-friendly nanodots but also provides multifunctional nanomaterials for pollutant detection, biomedical imaging, and environmental monitoring.

However, despite the remarkable advantages of CHM-CDs in analytical detection and the resource utilization of medical waste, the complexity of and variability in their internal composition still pose challenges to large-scale sustainable application. Some studies have attempted to synthesize carbon dots using single components derived from Chinese herbal medicine, yet this approach inevitably incurs high costs. In response, researchers have focused on enhancing the refinement of raw materials to improve the performance and reproducibility of CDs. For instance, one study precisely selected germinated cumin seeds as the carbon source via a hydrothermal method to prepare CDs. The resulting product (CDs) exhibited a higher quantum yield than those synthesized from seed coats or whole seeds and was successfully applied in the detection of rutin in Pueraria lobata, rutin tablets, and capsules [105]. This work underscores the importance of the precise classification of natural carbon sources in the preparation of CDs and offers a feasible strategy. On the other hand, traditional chemical separation methods can also purify the raw material composition to obtain CDs with better reproducibility. For example, specific extraction techniques can be employed to obtain certain types of precursor substances for subsequent CD synthesis [106]. Overall, there remains considerable room for improving the reproducibility of CHM-CDs. It is well recognized that high-quality, high-purity CDs are closely linked to pure raw materials, mature synthesis methods, and efficient purification processes.

### 5.2. Enhancement or Improvement in Pharmacodynamic Effects

An emerging line of research has revealed that CHMs and their active ingredients may display enhanced pharmacological efficacy and altered properties when transformed into QDs. This phenomenon provides new opportunities for the advanced utilization of CHM resources. Zhao et al. [107] synthesized CDs via a one-pot hydrothermal method using Forsythia extract as the carbon source. Compared with the crude extract, the CQDs exhibited significantly stronger antibacterial activity, with demonstrated practical value in wood preservation. Moreover, the inherent fluorescence of the CQDs enabled the real-time tracking of their distribution within wood tissue, suggesting dual utility as both a preservative and an imaging probe. Interestingly, carbonization—a traditional processing method widely employed in CHMs—has recently been linked to the in situ formation of CDs, offering a modern nanotechnological explanation for the altered pharmacological properties observed in charred herbal medicines [108]. For example, CDs derived from charred licorice exhibited therapeutic efficacy and safety in a mouse model of acute alcoholic gastric ulcer [109]. Similarly, CDs prepared from charred Platycodon grandiflorus displayed low toxicity in hyperbilirubinemia models, where they reduced oxidative damage, inhibited the elevation of bilirubin and inflammatory factors, and improved antioxidant levels and survival rates, thereby showing promise for treating hyperbilirubinemia and its associated liver injury [110]. In addition, CDs prepared from charred Scutellariae Radix Carbonisata demonstrated significant anti-allergic effects [111].

Within CHMs, carbonization is a longstanding practice used to enhance therapeutic efficacy, particularly for hemostatic applications. Traditional examples include carbonized preparations of Panax ginseng, Sanguis draconis residues, and palm charcoal, which are clinically employed to manage hemorrhagic disorders [112]. Recent studies have confirmed that such charred herbal products inherently generate CDs during high-temperature processing. For instance, CDs prepared from the scorched pollen of cattail, lotus rhizome, and artichoke demonstrated hemostatic effects by modulating both intrinsic and extrinsic coagulation pathways, while also exhibiting anti-inflammatory activity and good biocompatibility [113]. These findings collectively indicate that the pharmacological benefits of carbonized CHMs may be at least partially attributable to the formation of bioactive CDs. After being converted into CDs, CHM and its extracts exhibit enhanced pharmacological effects. This improvement can be attributed partly to the ability of CHM-based CDs (CHM-CDs) to optimize the intrinsic properties of the herbal materials, such as solubility and particle size distribution. On the other hand, it also stems from the inherent advantages of using CHMs as CD precursors, their richness in bioactive compounds with inherent therapeutic effects. During the formation of CDs, the pharmacological activities of some of these bioactive components are preserved or even potentiated [114]. For instance, Han et al. [113] prepared CDs from four different herbal medicines using the same high-temperature pyrolysis method. These CDs not only demonstrated excellent biocompatibility but also promoted hemostasis through both exogenous and endogenous pathways. Through systematic characterization, it was further revealed that the hemostatic effect and underlying mechanism of these four types of CDs are closely related to their specific structures and surface functional groups.

**Figure 6:**
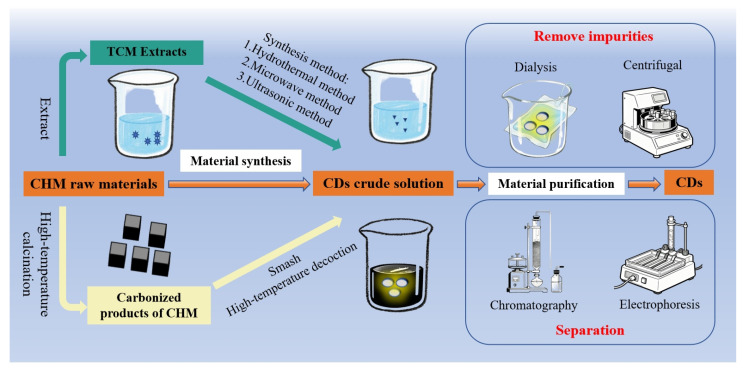
Key pathways for the preparation of CQDs from CHMs.

As summarized in Figure 6, two principal strategies are employed for CDs’ preparation from CHMs: (i) hydrothermal, microwave, and ultrasonic methods using herbal extracts as precursors, and (ii) high-temperature carbonization of raw or processed herbal materials. Regardless of the synthesis route, purification is a necessary step before biomedical applications. In fact, what we are presenting here is the currently mainstream method for preparing biomass carbon dots, namely the bottom-up approach [115]. This approach utilizes traditional Chinese medicinal materials as natural precursors. Through green processing methods such as hydrothermal treatment or high-temperature pyrolysis, the complex natural chemical constituents within these materials—including alkaloids, flavonoids, and polysaccharides—undergo a series of carbonization and spontaneous surface passivation reactions. These reactions encompass dehydration, polymerization, and aromatization, ultimately transforming the precursors into fluorescent and bioactive carbon dots. Notably, according to molecular fluorescence theory, precursors typically form numerous molecular fluorescent groups prior to CD formation. These groups lack the wavelength-dependent optical properties characteristic of CD emission. Such small molecules are termed fluorescent impurities, whose presence is also considered a primary cause of CD fluorescence release [116]. Therefore, purifying CDs is a crucial step when exploring their properties. Liu et al. [117] employed mixed solvents to extract CD solutions, discovering that CDs in water exhibited green luminescence while those in dichloromethane displayed red luminescence. Figure 6 illustrates two purification approaches: conventional removal methods (high-speed centrifugation and dialysis) and separation methods (chromatography, electrophoresis, extraction). The judicious application of these techniques holds significant value in enhancing CD quality [118].

The therapeutic promise of CHM-derived CDs extends well beyond traditional applications. Their small size, tunable optical properties, and excellent biocompatibility render them suitable for innovative clinical uses [114]. Beyond herbal sources, mineral drugs have also been explored as precursors for QD fabrication. Pu et al. [119] developed nano-andrographolide QDs (NR QDs) from Andrographis and further functionalized them with the arginine–glycine–aspartic acid (RGD) tripeptide to target tumor neovascularization. In vivo validation in a mouse tumor model revealed that NR@RGD QDs actively localized to tumor endothelial cells, where they inhibited angiogenesis and effectively blocked the nutrient supply to tumors. Such findings exemplify the translational potential of QDs derived from CHMs and related natural products, not only as therapeutic agents but also as multifunctional platforms that combine treatment, targeting, and imaging capabilities.

The therapeutic potential of CHM is attributed to the various functional groups present in its active ingredients, such as carboxyl groups, alcohols, phenols, and amines. These functional groups account for more than half of the components of CHM and have been proven to have pharmacological activity, resulting in its unique medicinal effects, including compatibility issues, the combination of multiple components, and multi-targeted actions [120]. Therefore, a comprehensive study and application of these active molecules or groups hold great promise for advancing traditional Chinese medicine as a viable treatment option.

**Figure 7:**
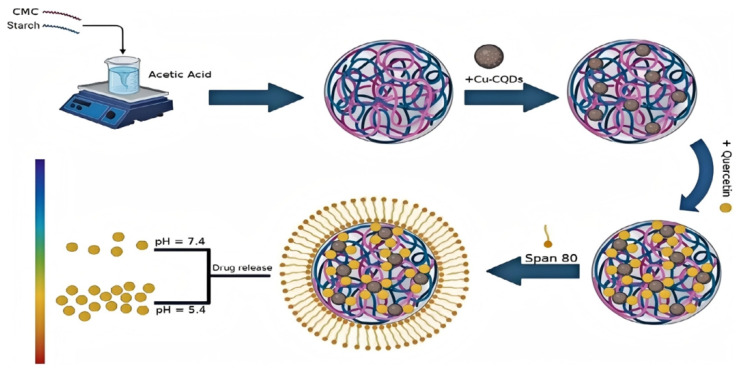
Schematic of the synthesis and targeted delivery of CMC/starch/Cu-CQDs@QC nanocarriers [122].

The active ingredients of CHMs can also be incorporated into or directly prepared as QDs, thereby enhancing their pharmacological efficacy and targeting. Curcumin, a well-known natural anti-tumor compound, suffers from poor solubility and low bioavailability, limiting its therapeutic potential. To address this, researchers developed a nanocarrier system composed of chitosan, alumina, and CQDs, forming an interconnected structure capable of capturing curcumin. This strategy significantly improved drug loading and encapsulation efficiency. Cytotoxicity and activity assays demonstrated that the nanocarriers exhibited stronger cytotoxic effects against tumor cells compared with free curcumin. Moreover, due to the pH-responsive nature of the carrier, curcumin could be delivered more selectively to tumor tissues, reducing the required dosage and minimizing side effects [121]. As shown in Figure 7, this study vividly demonstrates the application of QDs in the targeted delivery of CHMs. Quercetin (QC), a potent flavonoid with potential therapeutic applications, faces challenges due to its hydrophobicity and poor solubility. This approach proposes a novel method to enhance QC’s solubility and stability by integrating it with copper–carbon quantum dots (Cu-CQDs) into pH-responsive carboxymethyl cellulose (CMC) and starch-based hydrogel nanocomposites. The nanocomposite exhibits excellent drug loading capacity (47.00% ± 0.45) and encapsulation efficiency (86.25% ± 0.75), improving QC delivery while minimizing adverse reactions and demonstrating strong selectivity toward cancer cells [122].

Similarly, sanguinarine (SAN), a plant-derived alkaloid with antibacterial properties, was loaded onto zwitterion-modified Ta_4_C_3_ MXene QDs to produce the antibacterial agent (SAN@AHEP@Ta_4_C_3_). In vitro and in vivo experiments confirmed its excellent antibacterial activity against *Staphylococcus aureus* and *Escherichia coli*, while also promoting wound healing in mice without causing toxicity to normal tissues or organs. Importantly, drug release was accelerated in acidic environments and further enhanced under near-infrared (NIR) irradiation. The QD-based system exhibited outstanding photothermal conversion ability, enabling the synergistic combination of chemotherapy and photothermal therapy to achieve efficient antibacterial effects [123]. QDs derived directly from CHM components can inherit bioactivity from their precursors. A recent study synthesized red/NIR-I fluorescent CQDs from rhubarbic acid, doped with L-arginine. These CQDs displayed reactive oxygen species (ROS) scavenging activity and demonstrated colon-targeting capability in colitis models. In addition to improving the solubility and bioavailability of rhubarbic acid, the CQDs enabled a transition from intraperitoneal to intravenous administration and facilitated the fluorescence imaging of inflamed colon tissue [124]. Collectively, these examples highlight the dual advantages of CHM-based QDs: they not only improve the pharmacokinetic limitations of poorly soluble natural products but also integrate therapeutic efficacy with diagnostic imaging, paving the way for multifunctional nanomedicine platforms in CHM modernization.

## 6. Summary and Outlook

QDs have emerged as versatile tools in CHM research, offering unique optical and catalytic properties that enable the sensitive detection of active ingredients, exogenous pollutants, and mycotoxins, as well as the effective photocatalytic degradation of environmental contaminants. CHMs and their by-products also serve as sustainable precursors for QD synthesis, generating eco-friendly nanomaterials with applications in pollutant sensing, bioimaging, and therapeutic enhancement. Notably, CDs derived from herbal sources can improve the solubility, bioavailability, and pharmacological activity of CHM components, while enabling multifunctional platforms for diagnosis and therapy.

Current research on quantum dots (QDs) in TCM highlights critical areas for improvement. For instance, in developing QD sensors for CHM contaminants, researchers often overlook the interference from complex herbal matrices. In reality, many key organic constituents—such as coumarins and flavonoids—exhibit fluorescent properties. This oversight may lead to false positives or signal masking when testing real samples. We believe that in the field of CHM research, QD sensors should not merely focus on sensor construction alone. Instead, the appropriate pretreatment and optimization of sensor structures should be conducted based on the specific research subjects. Another noteworthy issue is that when quantum dots are used in clinical applications, safety becomes a primary concern. Unfortunately, most existing studies on their safety remain confined to the cellular level, with insufficient research on their long-term toxicity to the human body and issues related to accumulation and metabolism. This may potentially limit further in-depth applications in this field.

Future progress in this field will hinge on three pivotal pillars: developing eco-friendly and scalable synthesis routes, deepening the mechanistic understanding to facilitate practical product translation, and implementing rigorous biosafety evaluations to enable clinical adoption. Integrating QDs with innovative platforms—such as dual-mode sensing, ecologically relevant culture models, and AI-enhanced detection—will be crucial to bridge the gap between laboratory research and real-world application. The strategic convergence of traditional Chinese medicine with cutting-edge nanotechnology positions QDs to play a transformative role in modernizing TCM, promoting its sustainable development, and advancing its global recognition.

## Figures and Tables

**Table 1:** Common QDs used for active ingredients detection in CHMs.

Types of QDs	Target Compound	Mechanism of Detection	Synthesis Method	Limit of Detection	Reference
CDs	Hyperin	Photo-induced electron transfer (PET) between hyperin and CQDs	Hydrothermal method	78.3 nM	[32]
Polyaniline-functionalized graphene QD-modified glassy carbon electrodes	Calycosin	QD composite-modified glassy carbon electrodes enhance the electron transfer rate at the sensor surface	Ultrasonic treatment	9.8 μM	[33]
N, S co-doped CDs	Baicalein	Static bursting of CDs with baicalein	Hydrothermal method	0.21 μM	[34]
CDs	Morin	Existing intramolecular filtering effect (IFE) between morin and CDs	Microwave heating method	0.12 μM	[35]
N-CQDs	Myricetin	Existing IFE between myricetin and CQDs	Microwave heating method	18.4 nM	[36]
Molecularly imprinted polymer-modified L-cysteine-modified Mn-doped zinc sulfide QDs	Celastrol	PET occurs between QDs and celastrol	Hydrothermal method	35.2 nM	[37]
QDs modified with beclomethasone nucleic acid aptamers	Beclomethasone	Recovery of burst fluorescence due to competitive binding of beclomethasone to aptamers	Hydrothermal method	0.1 μM	[38]
Molecularly imprinted polymer-modified ratiometric fluorescent probe	Aristolochic acid I	PET between aristolochic acid I and QDs	Solvothermal reaction	0.45 μM	[39]
Molecularly imprinted polymer-modified FeS_2_ QDs	Aconitine	PET between aconitine and ratiometric fluorescent sensors	Solvothermal reaction	24 nM	[40]
SiO_2_-encapsulated green perovskite quantum dots and red perovskite quantum dots loaded with MIPs forming dual-quantum dot nanospheres	Rhein	Rheum emodin interacts with composite materials to form new complexes, triggering static quenching	Hot injection method	1.90 nM	[41]
CQD-Ru/multi-walled carbon nanotubes	Kaempferol	Specific binding of Kaempferol to composite materials	Hydrothermal method	24 nM	[42]

**Table 2:** QD sensing technologies for pollutant detection.

Detection Mechanism	Type of QDs	Target Analyte	Pollutant Category	Core Principle	Reference
Enzyme Inhibition	SiQDs	Methyl parathion	Pesticide Residue	Based on the inhibition of tyrosinase by methyl parathion, preventing dopamine generation and thereby affecting QD fluorescence.	[46]
Direct Interaction	TGA@Mn-ZnS QDs	Cypermethrin	Pesticide Residue	Pesticide forms ester adducts with carboxyl groups on the QD surface, leading to fluorescence quenching.	[49]
Competitive Binding/FRET	CdTe@ZnS QDs	Chlorpyrifos	Pesticide Residue	Degradation products of the pesticide displace DZ on the QD surface, disrupting FRET and restoring the QD fluorescence quenched by DZ.	[50]
Aptamer Recognition	GQDs	Acetamiprid	Pesticide Residue	Binding of the aptamer to the pesticide causes separation of the complementary strand, inducing changes in the QD signal.	[54]
Aptamer Recognition	Nitrogen-doped carbon dots (N-CDs)	Cd^2+^	Heavy Metal Ion	Binding of the aptamer to Cd^2+^ causes fluorescence quenching.	[63]
Direct Interaction	CDs	Hg^2+^	Heavy Metal Ion	Hg^2+^ binding to surface carboxyl groups causes QD aggregation and electron transfer, leading to quenching.	[64]
Aptamer Recognition	Ag_2_S QDs	Zearalenone	Mycotoxin	The specific binding of aptamers and toxins blocks electron transfer from ascorbic acid (AA) to the Bi_2_S_3_/BiOCl-Ag_2_S composite, decreasing photocurrent.	[61]
Antibody Recognition	ZnCdS@ZnS QDs	Aflatoxin B_1_	Mycotoxin	QDs generate excited states (QDs*) via electron transfer, producing ECL emissions. Steric hindrance from AFB1 binding decreases the conductivity of the modified electrode.	[72]
Dispersion and Electron Transfer	Boron-doped graphene quantum dots (B-GQDs)	Phthalate esters	Plasticizer	Plasticizers affect the dispersion stability of B-doped graphene QDs in different solutions, accompanied by changes in their fluorescence signal.	[75]
Indirect Interaction	N-CDs	Sulfite	SO_2_ Derivative	Redox reaction between Cr(VI) and HSO_3_^−^ reduces Cr(VI) to Cr(III), inhibiting the inner filter effect (IFE) caused by Cr(VI) and restoring QD fluorescence.	[76]
Direct Interaction	MNP-SiO_2_-CdTe QDs	Enrofloxacin, etc.	Antibiotics	FRET and electron transfer.	[80]

**Table 3:** Application of QDs in the degradation of some pollutants.

Photocatalytic Material	Pollutants	Results of the Study	Light Source Type	Reference
Polyethyleneimine and polyethylene glycol-modified GQD	Methylene blue (MB)	Almost completely degraded after 4 h of irradiation	300 W Xe lamp	[88]
Perovskite QDs	Methyl orange	QDs can decompose the MO solution into a colorless solution within 100 min	Visible-light irradiation	[89]
BNQD-modified bisphenol S-doped g-C_3_N_4_ nanosheets	Sulfadimethoxine	100% degradation efficiency in 60 min	300 W Xe lamp	[85]
Cu-doped CQDs loaded on two-dimensional Ni-MOL (one of MOF, Ni-metal organic framework) to construct novel two-dimensional photocatalysts	Tetracycline	The degradation rate reached about 93.5% within 60 min under visible-light irradiation	300 W Xe lamp	[90]
CdTe/ZnSe core–shell QDs were synthesized successfully by ultrafast, one-pot, and simple microwave method in aqueous media	Methylene orange (MO)	The degradation rates of MO under UV light irradiation were 79% and 70%	UV and daylight simulation lamps	[91]
SnO_2_ QDs were prepared from SnCl_2_-2H_2_O in aqueous solution using SnCl_2_-2H_2_O as a raw material and CH_4_N_2_S as a catalyst	Octane	Under optimal conditions, 91.9% of the octane was degraded after 48 h of light exposure	150 W high-pressure mercury lamp	[92]
Sulfur-doped graphene quantum dots (SG-QDs) were first stabilized on the surface of ZnS semiconductor nanoparticles to construct the core–shell structure ZnS-SG QDs, and then modified with Ag_2_S nanoparticles	Diazinon	Degradation of more than 99% of diazinon in 60 min	60 W LED lamp	[93]
Construction of photo-Fenton reaction systems by modifying N-CQD on the surface of supramolecularly self-assembled carbon nitride and introducing Fe ions into the planar structure of carbon nitride	Ciprofloxacin (CIP)	76% degradation of CIP in 120 min	300 W Xe lamp	[94]
CdS QDs stabilized with 4-(2,2:6′,2-bis(terpyridinyl)-4′-yl)benzoic acid were loaded onto the surface of zeolitic imidazole skeleton	Arsenicals	Degradation of 93.12% arsenicals in 90 min under alkaline conditions	35 W LED lamp	[95]
Green synthesis of N-CQDs from citrus peels using microwave-assisted methods	Chlorpyrifos	91% chlorpyrifos degradation in 70 min	Daylighting (11:00 a.m. to 2:30 p.m.)	[96]
Construction of CDBHCF nanocomposites by anchoring carbon dots (CDs) and cobalt ferrite (CF) particles to boehmite (BH)	Tetracycline	92% degradation in 120 min under visible light	150 W Xe lamp	[97]

## Data Availability

Data is contained within the article.
